# Prevalence and shared risk factors of HIV in three key populations in Vietnam: A systematic review and meta-analysis

**DOI:** 10.1017/S0950268823001243

**Published:** 2023-08-01

**Authors:** Patricia Lee, Ashraf Docrat

**Affiliations:** 1 School of Medicine & Dentistry, Griffith University, Gold Coast, QLD, Australia; 2 Department of Medical Research, China Medical University Hospital, Taichung City, Taiwan

**Keywords:** female sex workers, HIV pooled prevalence, men who have sex with men, people who inject drugs, shared risk factors

## Abstract

This study aims to estimate the prevalence of HIV among each of the three key populations in Vietnam: people who inject drugs (PWID), female sex workers (FSW), and men who have sex with men (MSM) and quantify their shared risk factors for HIV infection through a systematic review and meta-analysis of recent literature (published in 2001–2017) in the relevant topics. A total of 17 studies consisting of 16,304 participants were selected in this review. The meta-analysis results revealed that the pooled prevalence estimates with 95% confidence intervals (CIs) among PWID, FSW, and MSM were: 0.293 (0.164, 0.421), 0.075 (0.060, 0.089), and 0.085 (0.044, 0.126), respectively. The findings also indicated that injecting drug use (OR: 9.88, 95%CI: 4.47–15.28), multiperson use of injecting equipment (OR: 2.91, 95%CI: 1.69, 4.17), and inconsistent condom use (OR: 2.11, 95%CI: 1.33, 2.90) were the shared risk factors for HIV infection among these population groups. The findings highlighted the importance of HIV prevention approaches to addressing the shared sexual and drug-related practices among the key populations in consideration of their overlapping social networks.

## Introduction

HIV infection remains a major global public health issue. According to the UNAIDS latest statistics, there were 1.5 million HIV infections and 650,000 AIDS-related deaths in 2021 alone [1, 2]. The extended coverage of antiretroviral therapy (ART) and international HIV prevention efforts have contributed to significant reductions in new HIV infections and AIDS-related deaths since 2000. In addition, effective HIV treatment programmes can prevent HIV transmission and enable people living with HIV to receive adequate treatments and continue to live well and productively [2]. The effectiveness of HIV treatments has resulted in the growing number of people living with HIV from 24.9 million in 2000 to 37.7 million in 2021 [2, 3]. Key populations including people who inject drugs (PWID), female sex workers (FSW) and their clients, men who have sex with men (MSM), transgender people, and their sexual partners are vulnerable to HIV infection. Over 94% of new HIV infections in Asia and the Pacific occur among key populations and their sexual partners [1, 2].

The first case of HIV in Vietnam was identified in 1990 and over the next 10 years it spread rapidly resulting in a country-wide epidemic that particularly impacted PWID. Vietnam has made significant progress in HIV control with substantial declines in new HIV infections and AIDS-related deaths since 2010 [1, 2]. The prevalence in the population overall (aged 15–49) has also stabilised at around 0.3% since 2015 [2]. However, reports by the Vietnamese Government and the World Health Organization (WHO) have indicated that the epidemic is concentrated primarily among three key populations: PWID, MSM, and FSW [2, 4, 5]. The prevalence in the three groups was projected to be 12.7%, 13.3%, and 3.1%, respectively, in 2021 [2]. When considering the implementation of UNAIDS 90–90-90 targets, the prevention of HIV transmission among key populations remains challenging. Despite over 50% of the people living with HIV in these three populations knowing their HIV status, the coverages of ART and HIV prevention programmes among them were below 30% (21.3%–26.5%), except 64% ART coverage in PWID [2]. These figures highlight the needs for more focused HIV prevention programmes prioritising the key populations.

Globally, PWID, FSW, and MSM face social and structural challenges including criminalisation, social exclusion, stigma, and discrimination which influence access to HIV prevention and increase the risk of acquiring HIV [1, 2]. They generally exhibit one or more higher-risk behaviours including selling sex, unprotected sex, drug use, and use of contaminated injecting devices. Many individuals belong to more than one key population and they can have shared social networks, which change the dynamics of the spread of HIV [6–9]. Evidence highlights that overlapping risk factors amplify the risk of HIV transmission among these key populations, such as injecting drugs among FSW and MSM, and PWID’s sexual links with FSW [4, 6]. Assessing the HIV prevalence in each key population and common risk factors among them can provide insight into the status of the HIV epidemic among these vulnerable populations and inform HIV prevention programmes.

Few systematic reviews have explored the risk factors among each of PWID, FSW, and MSM groups in the Vietnamese context. For example, Garcia et al. conducted a systematic review on HIV prevalence and risk behaviours among MSM and found that inconsistent condom use and low rates of regular testing were risk factors for HIV [10]. However, no studies have attempted to explore potential common behavioural risk factors among the three populations together in Vietnam. International studies on similar topics are also limited. A systematic review and meta-analysis by Malta et al. in Brazil identified injecting drug use and unprotected anal sex as common risk factors in FSW and MSM; while injecting drug use, use of contaminated injecting equipment, engagement in sex work, and sex among men were the key predictors of HIV infection among people who use drugs [11]. However, the study did not determine the prevalence of common HIV risk factors and estimate their associations with HIV among the key populations. Thus, the objectives of this study are to estimate the overall HIV prevalence and identify the prevalence of shared risk behaviours, their strengths of association with HIV infection among three key populations: PWID, FSW, and MSM in Vietnam through a systematic review and meta-analysis of published literature in the relevant topics. The findings will allow for a better understanding of HIV status and underlying connections among these populations and inform an improved HIV response addressing shared risk factors.

## Methods

As the trend of HIV in Vietnam has stabilised since the early 2000s (5, p.3), the literature search of this review focused on studies published from January 2000 to November 2018. This study followed the similar search and study selection strategies reported in the previous systematic reviews on this topic [6, 10, 11]. In addition, the PRISMA Statement for Quality Assessment (Supplementary Table S1) and Data Reporting [12] were used for study selection and appraisal. Due to the observational nature of studies concerning the relevant topics, other international guidelines including the Meta-analysis of Observational Studies in Epidemiology (MOOSE) and the TREND statement (Transparent Reporting of Evaluations with Non-randomised Designs) were also used to supplement the PRISMA Statement for quality assessment in regard to sampling and adjustments for confounding, especially the non-randomised aspects in the conduct of each study [13, 14].

### Search strategy

The literature search was conducted using five electronic databases: MEDLINE, PubMed, Scopus, Science Direct, and Google Scholar. All searches included the terms ‘risk factors’ or ‘associated factors’ or ‘correlated factors’ or ‘correlates’ or ‘risk behaviours/behaviors’; and ‘HIV’ (or ‘human immunodeficiency virus’ or ‘HIV/AIDS’); and ‘Vietnam*’ (or ‘Viet Nam’); as well as at least one of ‘men who have sex with men’, ‘female sex workers’, ‘women who sell sex’, ‘people who inject drugs’, ‘injecting drug users’, or ‘people who use injection drugs’. An example of search strategy is detailed in the Supplementary Material (Supplementary Table S1). The keyword search processes were applied to the various databases. All identified studies were imported into an EndNote reference management file. In addition, manual search was also performed to identify relevant publications through screening of references listed in the included studies and governmental and HIV organisation reports. Governmental and HIV organisational reports provided useful background information (such as the HIV situation in Vietnam) for this research, and they were identified through Google search and specific searches (e.g., HIV prevention programmes in Vietnam/Viet Nam) on the UNAIDS and WHO websites. The literature search was conducted with the assistance of a specialist at the Griffith University Library. The final search was completed at the end of November 2018.

### Inclusion criteria

The free full-text studies including open-access and institutional licensed publications were accessed through Griffith University’s databases. Peer-reviewed original studies which collected their data in Vietnam in relation to HIV in any of the three key populations were included as this review is only concerned with these populations. In order to ensure the applicability of the results of this review to the Vietnamese context, data collected from Vietnamese people living in a different country or from bordering countries were deemed ineligible. As the present review intended to quantify the effects of multiple risk factors on HIV serostatus, only studies including quantitative methods (both data collection and analysis) and using serological tests to determine the HIV status among the selected populations were considered. In addition, the included studies pertaining to at least one of the three key populations outlined above had to report multivariable analysis results to assess the independent association between each identified risk factor and HIV. The ‘risk factors’ in this review were determined if a positive association (with an adjusted odds ratio, OR > 1 reaching statistical significance) between each study factor and HIV was established in the included studies. The data on the identified risk factors (whether significantly associated with HIV or not) were extracted from all available studies included in this review to estimate the pooled effects for HIV in meta-analyses. The study factors ranged from sociodemographic variables, behavioural risk factors, and status of sexual transmitted infection to HIV-related perception and knowledge. As the definitions of behavioural risk factors varied across studies, the most common and broader definitions were applied. For instance, injection drug use was defined as ‘ever injected drugs’ which covered definitions from ‘injected drugs in last month’ to ‘injected drugs in the past 12 months’. Consistent condom use was defined as ‘always used a condom during sex (including anal sex) in the past 12 months’ (otherwise ‘inconsistent condom use’) to include those definitions based on more recent experiences such as consistent condom use in the last month, 3 months, or 6 months. Consistent/inconsistent condom use during annal sex was only specified in two MSM studies [7, 9].

### Exclusion criteria

Studies which did not determine the HIV status (as a study outcome) of the test subjects by serological test were eliminated in order to ensure the quality of outcome measurement. The serological test results could either be from blood samples taken by and analysed by the research teams or from official medical records using standard testing methods. Furthermore, all data could not be purely qualitative and at the very least, a quantitative component in mixed methods studies was necessary in order to quantify HIV prevalence and the association of common risk factors with HIV in meta-analysis. Finally, studies which were not published in English or where the full-text could not be accessed via Griffith University’s databases were also excluded.

### Study selection and data extraction

All the individual EndNote files were then merged and scanned for duplicates; and all duplicates were removed. Ineligible studies were eliminated from the combined EndNote library following title and abstract screening. Then, full-texts of the remaining publications were downloaded and assessed for eligibility based on the above-mentioned criteria. Data extracted for this review included authors, year of study, study region/city, study design, data collection and analysis methods, HIV prevalence (with 95%CI), and risk factors identified by the included studies. The data of identified risk factors were extracted from available studies. The odds ratio data (with 95%CI) determining the association between each risk factor and HIV status were extracted from the table reporting the multivariable results (main findings).

### Statistical analysis

All the extracted data such as prevalence, odds ratio, as well as sample size of each individual study were entered into the Comprehensive Meta-Analysis (CMA-v.3) datasheets. The CMA software was used to perform the analysis of pooled HIV prevalence estimate of each key population and pooled prevalence of common HIV risk factors across key populations, and pooled effect estimates (odds ratios) of common risk factors for HIV status. The I^2^ Index was assessed to determine the degree of heterogeneity among studies. According to Higgins & Thompson, levels of heterogeneity were defined as low, medium, and high using corresponding I^2^ values of 25%, 50%, and 75%, respectively [11, 15]. A random-effects model was chosen instead of a fixed-effects model when the calculated I^2^ value in each meta-analysis reached ≥75%. If studies included several subsamples, various levels of prevalence in the subsamples were used for the aggregated prevalence estimate. Sensitivity analyses were conducted to assess potential sources of heterogeneity due to the inclusion of specific studies. Funnel plots and associated tests (Duval & Tweedie’s method, Begg’s rank test, and Egger’s regression intercept) were used to examine potential publication bias. Statistical significance was established based on a *P* value <0.05.

## Results

**Figure 1.**
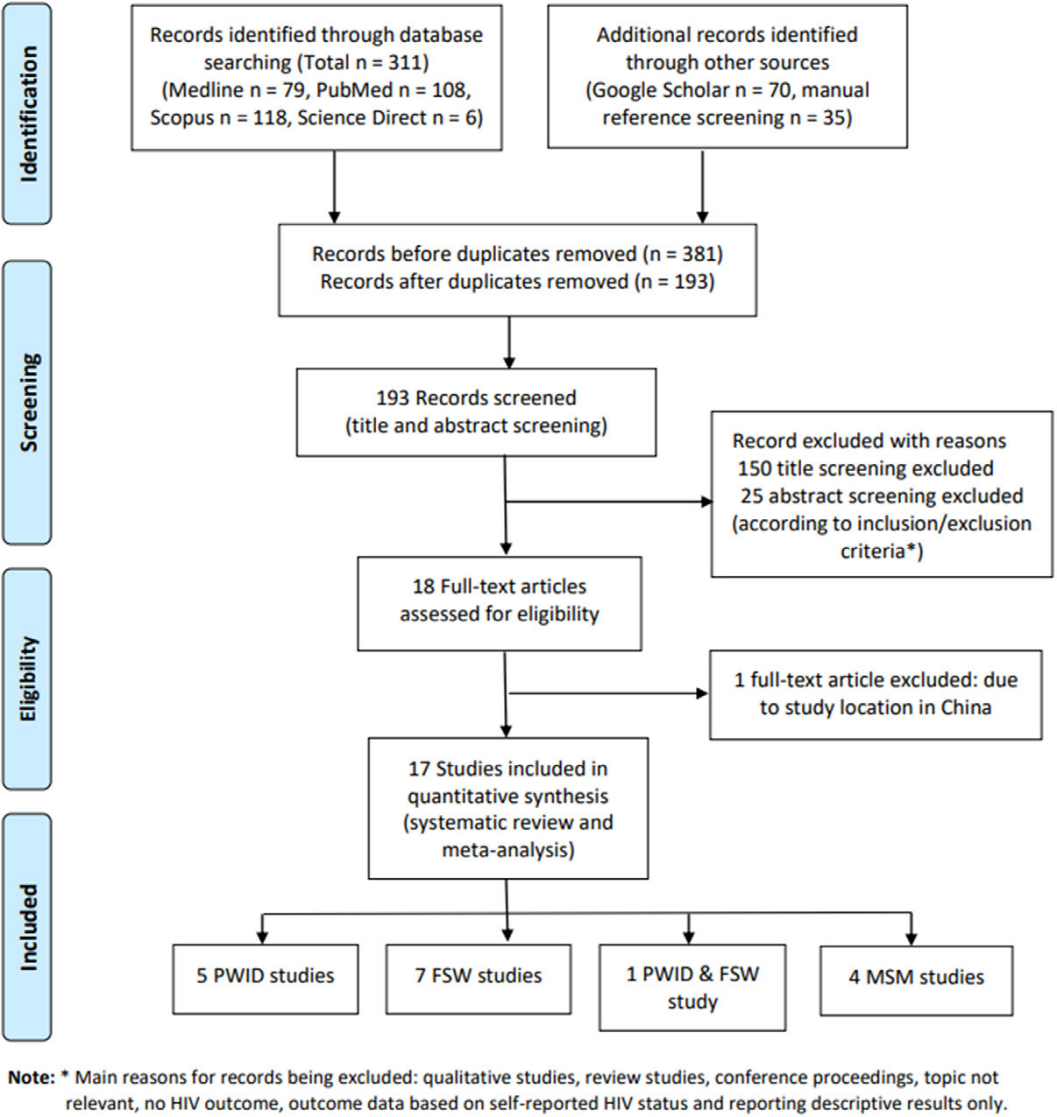
PRISMA flow diagram – Literature screening process.

Figure 1 details the change in the number of included records at each stage of the screening process. At the end of the study selection, seventeen publications [7–9, 16–29] were included for this systematic review and further meta-analysis.

The summary of the key extracted findings is presented in Table 1. Of the included studies (publication years ranging from 2001 to 2017), five focused on PWID, seven on FSW, four on MSM, and one on both PWID and FSW, with a total of 16,304 participants (3,084, 9,073, and 4,147 respondents in the three subgroups, respectively). Due to overlapping risk behaviours, some respondents identified as one key population group may actually be members of multiple population groups. All the PWID studies were conducted prior to 2005 (1997–2004), while five FSW studies and one MSM study were carried out after 2005. It is noted that 6 studies [7, 19, 20, 26, 27, 29] were solely conducted in large cities such as Ho Chi Minh City (HCMC), Hanoi and Hai Phong, and the remaining studies were generally well geographically spread across the country.

**Table 1.** Characteristics of the included studies, HIV prevalence and behavioural risk factors in three key populations

References	Year of study	Study region/city	Target population (sample size)/study design	HIV case ascertainment (HIV prevalence)	Significant behavioural risk factors
Go et al. [16]	2003	Bac Ninh Province (semiurban)	PWID (Male PWID aged 18–45) (299) Community-based cross-sectional	Serological Test (42.8%)	Duration of injection: OR: 4.51 (2.39, 8.50) Pooling money to buy drugs: OR: 3.70 (1.99–6.90) Number of new syringes (>^1^) you get at a time: OR: 0.36 (0.19–0.67)
Quan et al. [17]	2004	Bac Ninh Province	PWID (309) (1) Cross-sectional (2) Matched case–control study	Serological Test (42.4%)	Sharing drugs: Frontloading, Yes OR: 2.75 (1.17–4.33)
Nguyen et al. [8]	2002–2004	4 border provinces: Lai Chau, An Giang, Dong Tap, and Kien Giang	PWID (2023) FSW (1391) Cross-sectional	Serological Test PWID: 4–36% Street-based FSW: 0–24.3% Karaoke-based FSW: 0–16.5%	PWID sample Multiperson use of injecting equipment: OR: 7.3 (2.3–12.3) Having sex with non-regular partners or FSW: OR: 3.4 (1.4–8.5) FSW sample Inconsistent condom use: OR: 5.3 (2.4–8.2) Injecting drug use: OR: 7.8, 95%CI not available Mobility (working in other provinces or abroad): OR: 3.0, 95%CI not available
Tran et al. (18)	2002	Long-an province	PWID (aged 14–29) (248) Cross-sectional	Serological Test (32%)	Injecting in another city: OR: 1.69 (0.92, 2.90) Multiperson use of needles: OR: 1.91 (0.92, 3.96)
Nguyen et al. [19]	1999	Hai Phong	PWID (319) Cross-sectional	Serological Test (74%)	Frequency of drug use: 31 times per month, OR: 2.37 (1.04, 5.42) Needle sharing: Yes, OR: 4.12 (1.82, 6.42)
Nguyen et al. [20]	1997–1998	Ho Chi Minh City (HCMC)	PWID (518) On the street (218) In a rehab centre: (300) Cross-sectional	Serological Test PWID in the street: 12% (1998 data) PWID in the rehab centre: 13.3% (1997 data)	Street sample Being in the street OR: 2.07 (1.04, 4.17) Sharing drug pots: OR: 2.20 (1.23, 3.17) Rehab sample Being injected by drug dealers: OR: 2.90 (1.54, 5.46)
Nguyen et al. [21]	2014	Ba Ria- Vung Tau (Southern province)	FSW (aged18+) (420) Community-based cross-sectional	Serological Test (2.6%)	Clients injecting drugs: Yes, OR: 10.26 (1.65, 63.88) Having anal sex (past month) Yes, OR: 5.84 (1.04, 32.78)
Le et al. [22]	2009	10 provinces: 4 northern; 2 central; and 4 southern provinces	FSW (aged18+) (5298) Cross-sectional	Serological Test HIV rates raging 0.3–23% (FSWs in large cities: >10%)	High HIV provinces Drug use: Non-injecting drugs, OR: 1.78 (1.16, 2.74) Injecting, OR: 3.44 (2.32, 4.56) Consistent condom usage: Yes, OR: 0.71 (0.52, 0.98) Low HIV provinces Drug use: Injecting drugs, OR: 22.05 (12.00, 32.10) Inconsistent condom use: OR: 1.11 (0.694, 1.526) Negotiate sex on street (street-based FSW): Yes, OR: 1.81(1.11, 2.95)
Tran et al. [23]	2006–2007	5 provinces in Mekong Delta Region): Ben Tre, Hau Giang, Kien Giang, Tien Giang, and Vinh Long	FSW (1996) - Street-based (SSW): 339 - Establishment-based (ESW): 1657 Cross-sectional	Serological Test (Average: 2.1%) -SSWs:3.8% -ESWs: 1.8%	Inconsistent condom use: OR: 2.08 (0.97, 3.19)
Nguyen et al. [24]	2003	Soc Trang province (in Mekong Delta)	FSW (406) *395 provided biological samples Cross-sectional	Serological Test (3.3%)	Ever using illicit drugs: Yes, OR: 87.3 (10.1, 164.5) Direct sex work: Yes, OR: 15.1 (1.14–200.4) Contraceptive method: Withdrawal: OR 0.07 (0.006–0.90)
Thuong et al. [25]	2002–2003	5 border provinces: Lai Chau, Quang Tri, Dong Thap, An Giang, and Kien Giang	FSW (911) Cross-sectional	Serological Test (Average: 4.5%) Prevalence ranging 1% -7% in different provinces	Number of clients: ≥ 9/week, OR: 2.80 (1.19, 6.59)
Tran et al. [26]	2002	Hanoi	FSW (400) Cross-sectional	Serological Test (12%)	Borrowing used injecting equipment: Yes, OR: 32 (9.2, 54.8) Condom use: not always with regular clients, OR: 7.7 (2.5, 12.9)
Nguyen et al. [27]	2000	Ho Chi Minh City (HCMC)	FSW (398) Cross-sectional	Serological Test (16.3%)	Injecting drug use: OR: 101.30 (33.55, 169.05)
Nguyen et al. [28]	2010–2012	Southern Vietnam (8 provinces): Tay Ninh, Dong Nai, Ba Ria-Vung Tau, Ben Tre, in Vinh Long, Dong Thap, Hau Giang, and Soc Trang	MSM (2768) Cross-sectional	Serological Test (Average: 2.6%) Rates ranging 0% - 8.64% in different provinces	Recreational drug use: Previously, OR: 7.37 (2.22, 24.52) Currently inhaling, OR: 19.29 (4.60, 80.92) Injecting drug use: Currently injecting, OR: 63.58 (28.20, 98.96) Types of drug use (ref. never used) ATS, OR: 28.87 (5.10, 163.54) Heroin, OR: 48.16 (25.23, 91.90) Sexual role: Receptive, OR: 0.28 (0.13, 0.62) Consistent condom use Frequently, OR: 0.07 (0.01, 0.09) Always, OR: 0.42 (0.08–2.22) Engaged in sex with foreigners in last 12 months: Yes, OR: 9.24 (1.83, 46.64) Consumed alcohol before anal sex in past 3 months: Sometimes, OR: 0.15 (0.06, 0.34)
Le et al. [7]	2009–2010	Ho Chi Minh City (HCMC)	MSM (399) Cross-sectional	Serological Test (14.8%)	Sexual partners injecting drugs: Yes, OR: 2.24 (1.06, 4.73) Having anal sex (past month): Yes, OR: 2.70 (1.10, 6.64)
Pham et al. [9]	2009	An Gian province	MSM (381): PWID-MSM (63) and non-PWID-MSM (318) Cross-sectional	Serological Test (14.8%) PWID-MSM (20.6%) Non-PWID-MSM (3.5%)	Injecting drugs: OR: 2.88 (1.12, 4.64) Unprotected sex with FSW (in past 12 months): Yes, OR: 4.88 (1.91, 7.85)
Nguyen et al. [29]	2004	HCMC	MSM (599) Cross-sectional	Serological Test (8%), ranging from 7% to 33%	Selling sex, OR: 8.61 (1.20, 61.69) Injecting drugs in last 12 months, OR: 30.35 (6.49, 54.21) Having more than 5 male sex partners in last month, OR: 2.43 (1.14, 5.17)

All of the included studies were cross-sectional, but one PWID study used a matched case–control design following a cross-sectional survey to collect survey data for multivariable analysis [17]. The study designs, data collection, and analysis methods used in the included studies are displayed in Supplementary Material (Supplementary Table S2).

### HIV prevalence

**Figure 2.**
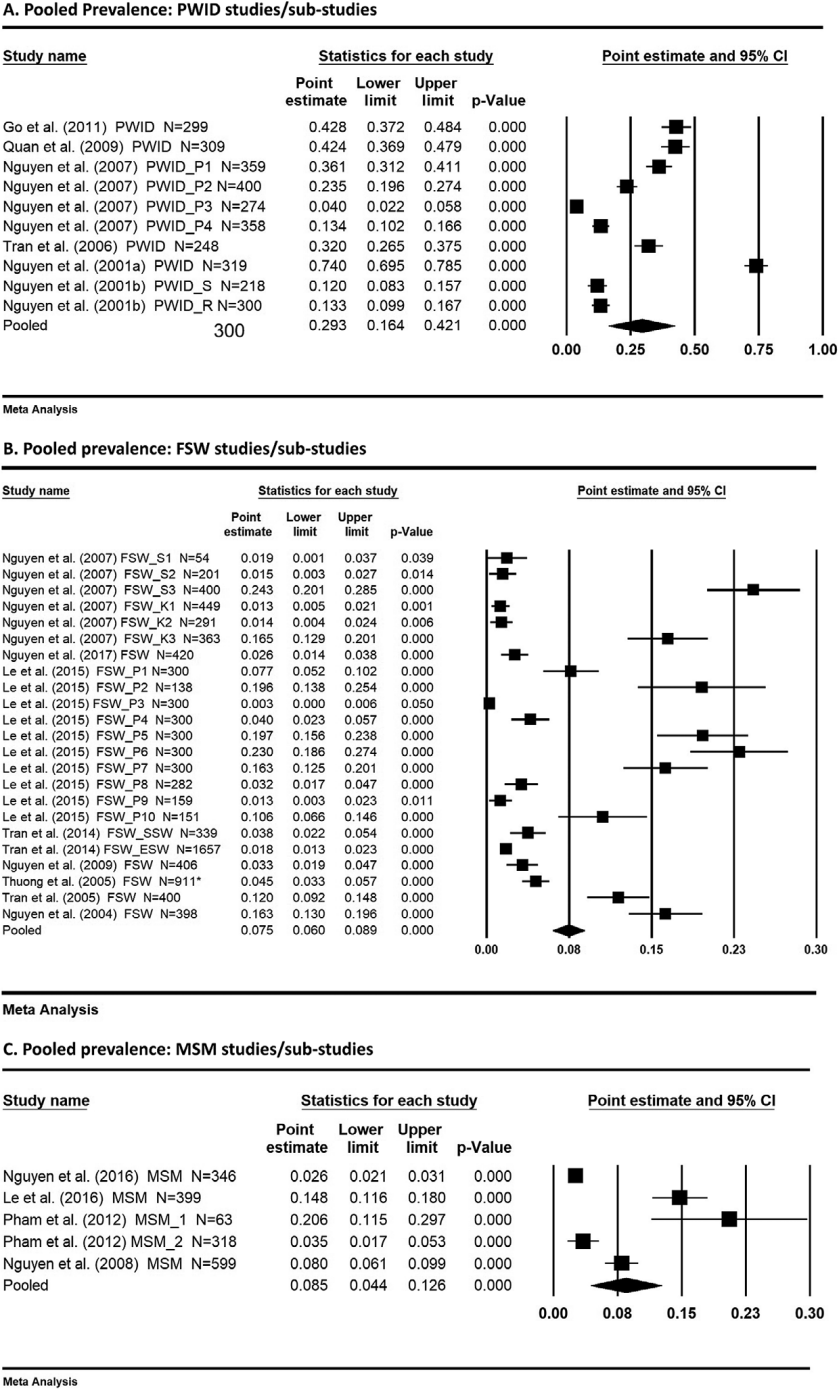
Estimated prevalence in different key populations (a–c). (a) Pooled prevalence: PWID studies/substudies. (b) Pooled prevalence: FSW studies/substudies. (c) Pooled prevalence: MSM studies/substudies. Note: 1. The size of each box is proportional to the weight (sample size) of the study, and the horizontal line through each box represents the 95% confidence intervals (95%CI) for the study measure; 2. The diamond shape represents the pooled estimate (prevalence) of the meta-analysis. The centre of the diamond is the point estimate, and the line ends reflect the 95%CI; 3. P1-P4 are subsamples drawn from different provinces; symbols ‘K, ‘S’, and ‘R’ are karaoke-, street-, and rehab centre-based subgroups. 4. MSM-1: MSM who use drugs, MSM-2: non-drug using MSM.

The prevalence of HIV across the key populations reported by the 17 studies was between 0 and 0.74. When broken down by population group, the prevalence of HIV in PWID varied greatly from 0.04 to 0.74 and it ranged from 0–0.243 and 0.026–0.33 in FSW and MSM populations, respectively. Some PWID studies [8, 19] included several subsamples. The sensitivity analysis indicated that removing a PWID substudy (with a very low prevalence of 4% [8]) did not significantly reduce the between-study heterogeneity. Therefore, all 10 PWID studies and substudies were included in the pooled prevalence estimate. The combined prevalence in PWID was 0.293 (95% CI:0.164, 0.421) in the final model (Figure 2a). Despite the symmetric funnel plot (data not shown), the test results suggested that potential risk of publication bias could not be ruled out (p value <0.05 for Egger’s regression). Following the same meta-analysis procedures, the overall HIV prevalence across 23 FSW subsamples was 0.075 (95% CI: 0.060, 0.089). The estimated prevalence among the five MSM studies (one study involving PWID and non-PWID MSM subsamples [9]) was 0.085 (95% CI:0.044, 0.126) (Figure 2b,c).

### Shared behavioural risk factors for HIV infection

#### Injecting drug use

**Figure 3.**
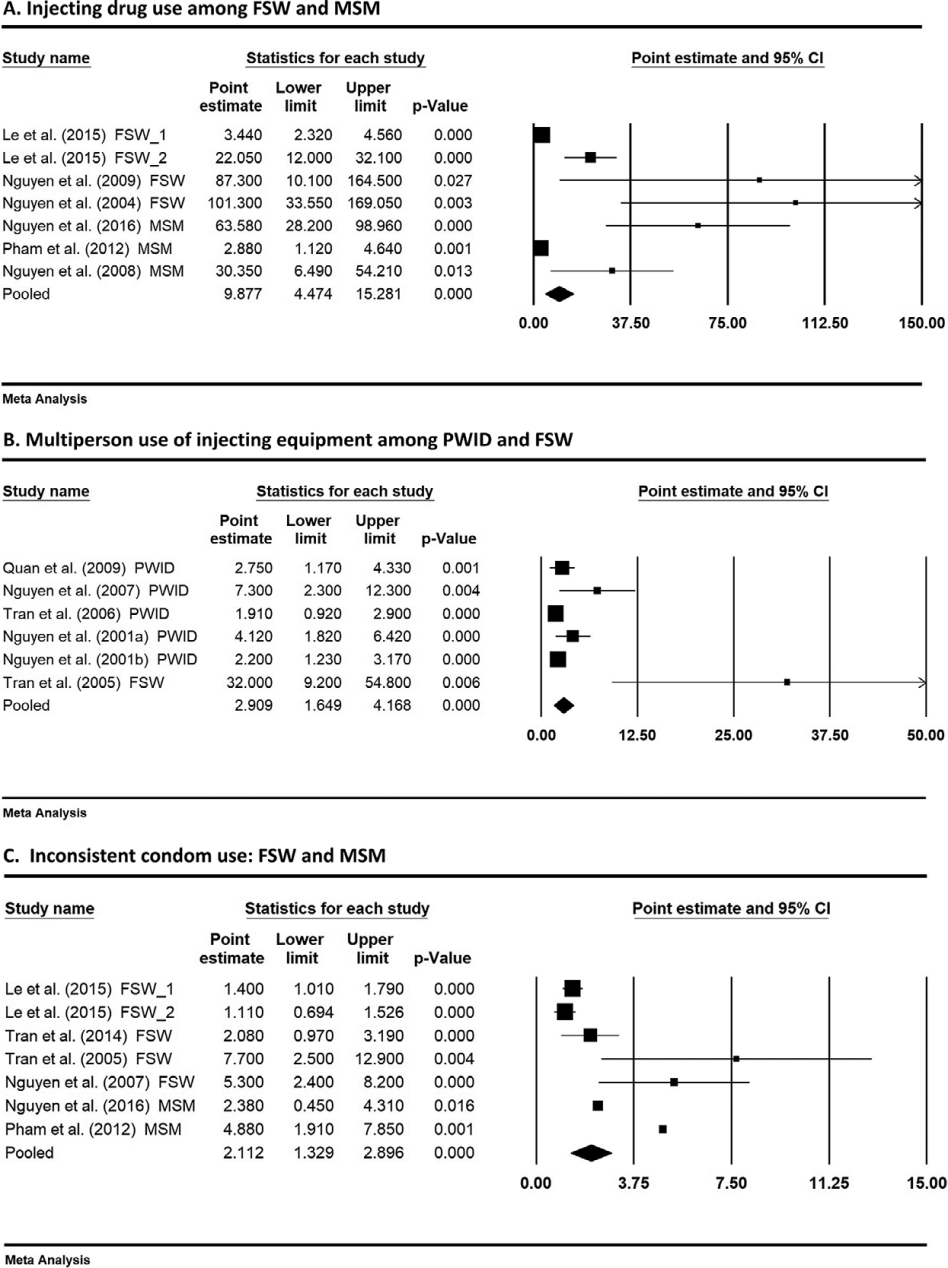
Pooled effect sizes of selected risk factors on HIV outcome. (a) Injecting drug use among FSW and MSM. (b) Multiperson use of injecting equipment among PWID and FSW. (c) Inconsistent condom use: FSW and MSM. The size of each box is proportional to the weight (sample size) of the study, and the horizontal line through each box represents the 95% confidence intervals (95%CI) for the study measure; 2. The diamond shape represents the pooled effect size (odds ratio) of the meta-analysis. The centre of the diamond is the point estimate, and the line ends reflect the 95%CI around the pooled odds ratio.

In the studies not involving PWID, injecting drug use was identified as a significant risk factor associated with HIV in seven studies (four FSW and three MSM studies). The estimated prevalence of injecting drug use was 0.093 (95% CI:0.050, 0.137) among FSW and 0.075 (95% CI:0.024, 0.127) among MSM (Supplementary Figure S1a,b). One FSW study by Nguyen et al. [8] was excluded from the meta-analysis, due to unavailable confidence interval for the risk estimate. Seven studies (two subsamples in Le et al. [22]) were included in the meta-analysis. A random-effects model (considering large heterogeneity among the included studies in the fixed model analysis) was chosen to estimate the pooled effect estimate (odds ratio) of injecting drug use on HIV prevalence in FSW and MSM populations. The result in Figure 3a showed a very strong association between injecting drug use and HIV. An almost 10-fold increase (OR: 9.88, 95% CI: 4.47, 15.28) in HIV risk was estimated, suggesting that injecting drug use significantly increased the risk for HIV among FSW and MSM.

In some studies, drug use of various types (injecting and non-injecting drugs) was investigated. For example, Nguyen et al. [28] in their MSM study specified drug use in the following details: previously using drugs, currently inhaling, injecting, using amphetamine-type stimulants (ATS), and currently using heroin. They found a very strong association between a variety of types of drug use and HIV.

#### Multiperson use of injecting equipment

Another important common risk factor identified by five studies was multiperson use of injecting devices (including needles/syringes) for injecting drug use. This risk factor was not unique to PWID studies but also identified in one FSW study [26]. While two studies [20, 26] used the term ‘equipment’ in relation to needles and related drug paraphernalia, one publication [20] described ‘sharing of drug pots’. Another study [17] described a particular method of drug taking: ‘frontloading’ injectable drugs, a process by which an injectable is mixed in one syringe and transferred to another. It is noted that the data on multiperson use of injection devices (and other drug use related behaviours) were commonly reported in PWID studies but largely unavailable in FSW and MSM studies. The synthesised prevalence of this behaviour among PWID was 0.223 (95% CI:0.082, 0.365) (Supplementary Figure S2a). As only one FSW study reported the percentage of multiperson use of injection equipment (20.3%), meta-analysis could not be performed to estimate the pooled prevalence in this population group. When pooling the data of PWID and FSW together for meta-analysis, the combined prevalence remained similar (0.22, 95% CI:0.098, 0.343) (Supplementary Figure S2b). The meta-analysis result of the pooled association of this risk factor with HIV is presented in Figure 3b. The pooled odds ratio from meta-analysis (using a random-effects model) was 2.91 (95%CI: 1.65, 4.17), indicating a significantly elevated HIV risk due to multiperson use of injecting devices in PWID and FSW populations. The result supported an increased risk of drug-related practice for HIV infection. As this risk behaviour was mainly available in PWID studies and only one FSW study reported it, the pooled effect should be interpreted with caution.

#### Inconsistent condom use

Data from 14 studies/substudies reporting percentages of consistent/inconsistent condom use were extracted to estimate the pooled prevalence in each of the key populations. Data on consistent condom use were converted to ‘inconsistent condom use’ using (1- reported percentage). The estimated prevalence of inconsistent condom use among PWID, FSW, and MSM was 0.511 (95% CI:0.419, 0.602), 0.393 (95% CI:0.266, 0.520), and 0.599 (95% CI:0.503, 0.696), respectively (Supplementary Figure S3a–c), indicating that inconsistent condom use was a risk behaviour shared among the three key populations. Inconsistent use of condoms was identified as a significant risk factor for HIV by four studies (three FSW and one MSM). In particular, three studies [8, 9, 26] reported a strong positive association (OR: 4.88, 5.30 and 7.7, respectively) between inconsistent condom use and HIV. Similarly, Le et al. 2015 [22] in their FSW study found an inverse association (OR: 0.71) between consistent condom use and HIV prevalence, indicating that consistent condom use is a protective factor against HIV. The association (OR) between ‘consistent condom use’ and HIV infection identified in Le et al. [22] was converted to ‘inconsistent condom use’ (where the odds ratio was inverted using 1÷OR), presenting a positive association with HIV. Figure 3c demonstrates a moderate pooled effect (OR: 2.11, 95%CI: 1.33, 2.90), meaning that inconsistent condom use among FSWs and MSM with their clients or sexual partners increased their HIV risk.

#### Other HIV risk factors

The other risk factors commonly identified in the systematic review were related to sexual practice and drug use among the key populations and their partners/clients. Two studies [7, 21] found an association between having sexual partners/clients who inject drugs and HIV prevalence in MSM and FSW (OR: 2.24 and 10.26, respectively). Engagement in anal sex was another HIV risk factor that was seen across subpopulations (MSM and FSW, with OR: 2.70 and 5.84) [7, 21]. Another common risk factor was being on the street identified in two studies for PWID and FSW [20, 22] with OR 2.07 and 1.81, respectively. Some studies included knowledge, perceived risk of HIV, and coinfection with other sexual transmitted infections (STIs) in their surveys. Sociodemographic risk factors such as age, marital status, education, and income were also commonly reported by many studies. However, different studies yielded inconsistent findings concerning the association between each of the sociodemographic factors and HIV status. Due to variations in the measurements of these variables across the selected studies, meta-analyses could not be performed to estimate the pooled effects. The results of systematic review on these variables are summarised in Supplementary Material (Supplementary Table S3).

## Discussion

The main findings of the meta-analyses suggested that the HIV prevalence in Vietnam was estimated to be 29.3%, 7.5%, and 8.5% in PWID, FSW, and MSM population groups, respectively. These figures were comparable with the data published by the ‘Vietnam AIDS Response Progress Report’ [4] in 2014, except for MSM. The report projected a decline of HIV prevalence from 30.4% in 2005 to 22% in 2013 among PWID but an increase from 4.9% to 5.3% among FSW and from 1.7% to 2.4% among MSM in the same years [4]. When comparing with the recent UNAIDS prevalence data, significant decreases were observed in PWID and female sex workers (12.7% and 3.1%, respectively, in 2021). However, an alarming increase was observed among MSM with HIV prevalence of 1.7% in 2005, 2.4% in 2009 [4], and 13.3% in 2021 [2]. Also, a systematic review published in 2012 suggested that HIV prevalence among MSM in Vietnam has been on the rise over time [10]. Our prevalence estimate (8.5%) for MSM using data from 2004–2012 seemed to have reflected the increase of HIV prevalence in this key population. In comparison with a similar study carried out in Brazil by Malta et al. [11], the HIV rates followed a similar pattern: 23.1%, 6.2%, and 13.6% for people who use drugs (PWUD), FSW, and MSM, respectively. The discrepancy in HIV rate in people who use drugs between the two countries could be due to their different sociocultural contexts and the inclusion of both PWID and PWUD (with a much lower HIV prevalence) in the Brazilian study.

Injecting drug use (among FSW and MSM), multiperson use of injecting equipment (PWID and FSW), and inconsistent condom use (FSW and MSM) were identified as shared behavioural risk factors significantly associated with HIV. The results also showed that the pooled prevalence of injection drug use among FSW (9.3%) or MSM (7.5%) was much higher than the estimated prevalence (0.27–0.53%) in Vietnamese adult population (aged 15–64) overall according to the United Nations’ World Drug Report 2022 [30]. Even though the result was synthesised based on data from FSW and MSM, injecting drug use remained a substantial independent risk factor for HIV among the key populations. For example, Vietnamese data showed that the odds of an FSW or MSM being infected with HIV are significantly higher among those who also report injecting drug use behaviour [4]. The recent UNAIDS data also reported that people who inject drugs have a 35 times higher risk of HIV infection than those who do not inject drugs [2]. This UNAIDS finding seemed to be much higher than our meta-analysis result (OR = 9.88 among FSW and MSM). This could be due to other factors mediating the relationship between injecting drug use and HIV status in FSW and MSM, and no data available from the PWID population in our meta-analysis. In addition to injection drug use, our meta-analysis found that over 20% of PWID and FSW were involved in multiperson use of injecting devices. As this behaviour significantly increased the odds of HIV infection (OR = 2.91) among these two populations, this finding further highlighted the need for addressing injecting drug use-related behaviours across the key populations. Furthermore, inconsistent condom use was identified as the most common overlapping risk factor (prevalence 40%–60%) among the three key populations. Our findings were in accord with the UNAIDS data published in 2021. The data showed that condom use was relatively low in PWID (41.9%) and MSM (65.2%) compared to FSW (89.6%) in Vietnam [2]. Thus, efforts to reduce barriers to condom use may be effective strategies to prevent HIV transmission, especially among PWID and MSM. The factors identified in this study were also consistent with the results of the above-mentioned Brazilian study [11], and some Vietnamese review studies focusing on each of the three populations [6, 10]. Ahmed and colleagues also found that sharing or reusing needles and other injecting equipment, sexual connections with sex workers/casual partners, and inconsistent condom use were common risk behaviours among PWID [6]. Another study on HIV risk factors in FSW (worldwide) showed that inconsistent condom use and intravenous drug use were significant risk factors associated with HIV in their meta-analysis [31].

To our knowledge, no studies employed meta-analysis to identify the shared risk factors among the three key populations. Most existing systematic review or meta-analysis studies either presented descriptive findings (such as percentages) or only focused on one key population. Importantly, the Vietnamese Government report noted that overlapping risk behaviours could intensify HIV transmission risks for FSW and MSM who also inject drugs [4]. Despite the lack of data consistently available among all the three key populations for our meta-analyses, our findings further provided quantitative evidence to support the importance of addressing overlapping risk behaviours and the interrelated sexual and drug use networks among the key populations [6, 8]. Due to legal reasons in the country, official data on the sizes of injecting drug use among the key populations are not available. Furthermore, the coverages of ART and HIV prevention programmes remained low among them (<30%, except 64% ART coverage in PWID). Given the elevated risks of injecting drugs and inconsistent condom use found in our meta-analyses and existing literature, the data indicated substantial unmet prevention need among these key populations.

The other common risk factors identified in our systematic review: having sexual partners/clients who inject drugs (MSM and FSW), engagement in anal sex (MSM and FSW) and being on the street (PWID and FSW), are mostly related to drug and sexual practices. HIV could be transmitted among these key populations and their partners/clients through sexual or drug-related contacts [6, 24, 32, 33]. It is crucial to reorient HIV prevention services to address the overlapping sexual and drug use networks among the three key populations. Close links and common higher risk sexual and drug use behaviours between PWID and FSW [6, 8, 26] and between PWID and MSM [9, 28, 29] are well documented in HIV studies based in Vietnam. However, evidence is limited to establish the potential overlapping transmission networks between FSW and MSM. Several Vietnamese MSM studies suggested that 22.1%–76.6% of MSM reported their sexual orientation as bisexual. In addition, these MSM engaged in either selling sex (gender of clients not specified) or having sex with FSW/ female clients (estimated prevalence: 6.6%–27%) [7, 9, 28, 29]. These risk behaviours are also compounded by sociocultural challenges such as legal concerns, stigma, and discrimination toward the key populations. The idea of ‘social-evils’ pervades Vietnamese culture [4, 34]. Those who engage in certain behaviours (such as sex work and illicit drug use) are subject to stigma and discrimination. These ‘social-evils’ largely stem from conservative, heteronormative, family-oriented views [7, 34]. In this context, men who have sex with men often tend to hide their sexual identity or decide to marry women to satisfy their families’ expectations and to avoid social stigma [7, 35]. The issues of stigma together with internalisation of their underpinning sociocultural norms can drive key HIV populations to become socially isolated, fear disclosing their HIV status, and impede them from accessing healthcare for testing, treatment, and other support services [7, 36–38]. The HIV prevalence among MSM shown in our meta-analysis together with an increasing trend of HIV prevalence demonstrated in official reports, and research data [2, 4, 10] have indicated gaps in meeting the HIV prevention needs in this population.

Recent studies suggested that understanding HIV transmission in mixing social networks among the key population groups could contribute to better HIV control and prevention [39, 40]. Further, interpersonal interactions in social networks and network characteristics (such as frequency of contact, social norms, close contacts, and social support) are crucial to understanding HIV risk factors and the spread of HIV infection. Social network approaches taking these into account could be helpful for developing more effective preventative strategies for key populations [39–43]. Williams and Dye [44] used an epidemic network modelling technique developed by Kato et al. [45] to explore the transmission of HIV among key populations (FSW, PWID, MSM) and their sexual partners or clients in Can Tho Province, Vietnam [44, 45]. The network model could be used to guide HIV prevention programmes such as needle exchange and condom distribution programmes. Interestingly, the model did not consider a direct link between FSW and MSM. Based on the findings of our review, future research is needed to extend the model to consider a potential link between these two groups considering their overlapping drug use and sexual networks and a worrying increasing trend of HIV rate in MSM. Social network approaches in addition to existing peer-driven strategies involving the key populations could provide a more comprehensive solution to addressing the common risk factors for HIV control and prevention in Vietnam [46–48]. A systematic review study found that some social network-based interventions (SNIs) have reached a greater proportion of key populations through peers chosen from these populations [42]. Peers are recruited and trained to disseminate health information and assist in delivering HIV prevention and treatment interventions among members from their drug and sexual networks in the community [42, 49, 50]. Peers play a crucial role as educators of HIV prevention information and health advocates in SNIs to create positive norms and social support for HIV-related services such as improving ART adherence and retention in HIV care, promoting safer sex and drug use behaviours, increasing engagement in needle exchange and addiction treatment programmes [42, 51–54]. These approaches also resonate with the recent UNAIDS’ call for supporting community-led HIV responses [3].

Drawing on the findings of this study, future research can explore the dynamics of social networks among the key populations to better understand the socio-cultural and economic influences that shape their overlapping risk behaviours. Future studies can also determine effective social network and peer-assisted approaches to reach the vulnerable populations and deliver HIV prevention and treatment services to them. The future directions for HIV prevention should consider programmes prioritised to the key populations and their sexual partners. These programmes can include improving access to HIV testing and services, strengthening the existing harm reduction programmes, and empowering peer educators to disseminate condoms and sterile injecting equipment and distribute health information for HIV prevention. In addition, stigma and discrimination remain the key barriers to accessing HIV services for key populations [4]. Continuing advocacy and social campaigns of antidiscrimination/stigmatisation are needed. The recent pilot project of take-home methadone maintenance therapy has demonstrated positive outcomes for PWID. Future programmes can scale up based on this initiative to develop ‘human rights-based integrated harm reduction and stigma reduction interventions’ [1]. Finally, HIV surveillance should consider standardising data collection tools (using consistent variable definitions) to monitor HIV trends and risk behaviours among key populations and inform HIV prevention strategies.

### Limitations

This study has the following limitations. Only quantitative publications reporting multivariable results were included in this review and meta-analysis study, while qualitative or descriptive studies were omitted. However, the large sample sizes of data in all three key populations might have helped minimise the potential impact of the exclusion of these studies. In addition, the estimated prevalence was validated with data published by Vietnamese Government and UNAIDS, showing consistent levels of HIV infection in the key populations. Another limitation was the inclusion of literature limited to English publications only. Furthermore, the data used to synthesise odds ratios of shared risk factors were not consistently available from all the included studies or all three key populations. In addition, the definitions of the common risk factors varied across studies, which could bias the meta-analysis results. The results might not be adequate to quantify the ‘common’ risk factors among these populations. It should be noted that the lack of data on the risk factor among existing works does not imply the factor is not prevalent among the population, only that it has not been measured or reported. The limitations mentioned above might have affected the meta-analysis findings. Despite these limitations, this study has demonstrated the strengths of estimating the pooled HIV prevalence among each of the three key populations and quantified the effects of the shared risk factors for HIV which are currently absent in existing literature.

## Conclusions

This systematic review and meta-analysis research have provided quantitative evidence to estimate the pooled HIV prevalence among each of the three key populations (PWID, FSW, MSM) in Vietnam and identified the shared risk factors among them. The findings have highlighted the importance of prioritising HIV interventions to the unique and perhaps at times overlapping needs of these populations and broader structural issues (such as stigmatisation) which impeded their access to HIV testing and treatment services. Future studies are recommended to explore the complex structure and dynamics of the overlapping networks to identify possible HIV transmission links among the key populations and their sexual partners to prevent HIV transmission.

## Supporting information

Lee and Docrat supplementary material Lee and Docrat supplementary material

## Data Availability

The extracted datasets for the meta-analysis are available on request from the corresponding author.

## Supplementary material

For supplementary material accompanying this paper visit http://doi.org/10.1017/S0950268823001243. click here to view supplementary material
