# Supplementary material for: Prevalence and shared risk factors of HIV in three key populations in Vietnam: A systematic review and meta-analysis
Source: Epidemiol Infect. 2023 Aug 1;151:e138. doi: 10.1017/S0950268823001243 (PMC10540180; doi:10.1017/S0950268823001243)
Supplement: Lee and Docrat supplementary material [file S0950268823001243sup001.docx]

***Epidemiology and Infection***

Manuscript title: Prevalence and shared risk factors of HIV in three key populations in Vietnam: A systematic review and meta-analysis

Authors: Patricia Lee and Ashraf Docrat

**Supplementary Material**

**Supplementary Table S1** Search terms/keywords used for literature search using different electronic databases by domain (using PubMed as an example)

| **PubMed search**  Search conducted in November 2018 |
| --- |
| **HIV domain** (total records: 104,297)  (HIV) OR (human immunodeficiency virus) OR (HIV/AIDS)  **Filters:** from 2000 – 2018, free full-text, English language. |
| **AND** |
| **AND Country domain** (total records: 7,700)  (Vietnam*) OR (Viet Nam)  **Filters:** from 2000 – 2018, free full-text, English language. |
| **AND** |
| **AND Key population domain** (total records: 78,802)  (men who have sex with men) OR (female sex workers) OR (women who sell sex) OR (inject drug users) OR (people who inject drugs) OR (people who use injecting drugs)  **Filters:** from 2000 - 2018, free full-text, English language. |
| **AND** |
| **AND Risk factor domain** (total records: 1,050,881)  (risk factors) OR (associated factors) OR (correlated factors) OR (correlates) OR (risk behaviors) OR (risk behaviours)  **Filters:** from 2000 –2018 , free full-text, English language. |
| **108 records identified** |

**Supplementary Table S2 PRISMA Quality Assessment Checklist**

| **Section/topic** | **#** | **Checklist item** | **Reported on page #** |
| --- | --- | --- | --- |
| **TITLE** | | |  |
| Title | 1 | Identify the report as a systematic review, meta-analysis, or both. | p.1, p.2, p.3 |
| **ABSTRACT** | | |  |
| Structured summary | 2 | Provide a structured summary including, as applicable: background; objectives; data sources; study eligibility criteria, participants, and interventions; study appraisal and synthesis methods; results; limitations; conclusions and implications of key findings; systematic review registration number. | p.2 |
| **INTRODUCTION** | | |  |
| Rationale | 3 | Describe the rationale for the review in the context of what is already known. | pp.3-5 |
| Objectives | 4 | Provide an explicit statement of questions being addressed with reference to participants, interventions, comparisons, outcomes, and study design (PICOS). | p.5 |
| **METHODS** | | |  |
| Protocol and registration | 5 | Indicate if a review protocol exists, if and where it can be accessed (e.g., Web address), and, if available, provide registration information including registration number. (PRISMA, MOOSE and TREND statement) | p.5 |
| Eligibility criteria | 6 | Specify study characteristics (e.g., PICOS, length of follow-up) and report characteristics (e.g., years considered, language, publication status) used as criteria for eligibility, giving rationale. | pp.7-8 |
| Information sources | 7 | Describe all information sources (e.g., databases with dates of coverage, contact with study authors to identify additional studies) in the search and date last searched. | p.6 |
| Search | 8 | Present full electronic search strategy for at least one database, including any limits used, such that it could be repeated. | Table S1 |
| Study selection | 9 | State the process for selecting studies (i.e., screening, eligibility, included in systematic review, and, if applicable, included in the meta-analysis). | pp.6-8, Figure 1 |
| Data collection process | 10 | Describe method of data extraction from reports (e.g., piloted forms, independently, in duplicate) and any processes for obtaining and confirming data from investigators. | p.8 |
| Data items | 11 | List and define all variables for which data were sought (e.g., PICOS, funding sources) and any assumptions and simplifications made. | p.7, p.8 |
| Risk of bias in individual studies | 12 | Describe methods used for assessing risk of bias of individual studies (including specification of whether this was done at the study or outcome level), and how this information is to be used in any data synthesis. | p.9, Table 1, Table S3 |
| Summary measures | 13 | State the principal summary measures (e.g., risk ratio, difference in means). | p.8 |
| Synthesis of results | 14 | Describe the methods of handling data and combining results of studies, if done, including measures of consistency (e.g., I^2^) for each meta-analysis. | p.9 |

Page 1 of 2

| **Section/topic** | **#** | **Checklist item** | **Reported on page #** |
| --- | --- | --- | --- |
| Risk of bias across studies | 15 | Specify any assessment of risk of bias that may affect the cumulative evidence (e.g., publication bias, selective reporting within studies). | p.9 |
| Additional analyses | 16 | Describe methods of additional analyses (e.g., sensitivity or subgroup analyses, meta-regression), if done, indicating which were pre-specified. | p.9 |
| **RESULTS** | | |  |
| Study selection | 17 | Give numbers of studies screened, assessed for eligibility, and included in the review, with reasons for exclusions at each stage, ideally with a flow diagram. | Figure 1 |
| Study characteristics | 18 | For each study, present characteristics for which data were extracted (e.g., study size, PICOS, follow-up period) and provide the citations. | Table 1, Table S3 |
| Risk of bias within studies | 19 | Present data on risk of bias of each study and, if available, any outcome level assessment (see item 12). | p.11,p.13 |
| Results of individual studies | 20 | For all outcomes considered (benefits or harms), present, for each study: (a) simple summary data for each intervention group (N/A) (b) effect estimates and confidence intervals, ideally with a forest plot. | Table 1 |
| Synthesis of results | 21 | Present results of each meta-analysis done, including confidence intervals and measures of consistency. | Figures 2-3 and Figure S1-S3 |
| Risk of bias across studies | 22 | Present results of any assessment of risk of bias across studies (see Item 15). | p.11, p. 13 |
| Additional analysis | 23 | Give results of additional analyses, if done (e.g., sensitivity or subgroup analyses, meta-regression [see Item 16]). | p.11 |
| **DISCUSSION** | | |  |
| Summary of evidence | 24 | Summarize the main findings including the strength of evidence for each main outcome; consider their relevance to key groups (e.g., healthcare providers, users, and policy makers). | pp.18-24 |
| Limitations | 25 | Discuss limitations at study and outcome level (e.g., risk of bias), and at review-level (e.g., incomplete retrieval of identified research, reporting bias). | p.24 |
| Conclusions | 26 | Provide a general interpretation of the results in the context of other evidence, and implications for future research. | p.25 |
| **FUNDING** | | |  |
| Funding | 27 | Describe sources of funding for the systematic review and other support (e.g., supply of data); role of funders for the systematic review. | N/A |

*From:*  Moher D, Liberati A, Tetzlaff J, Altman DG, The PRISMA Group (2009). Preferred Reporting Items for Systematic Reviews and Meta-Analyses: The PRISMA Statement. PLoS Med 6(7): e1000097. doi:10.1371/journal.pmed1000097

For more information, visit: **www.prisma-statement.org**.

Page 2 of 2

**Supplementary Table S3** Study designs, data collection and analysis methods used in the included studies.

| **Authors/ year of publication** | **Target population (sample size)** | **Study design** | **Data collection and analysis methods** |
| --- | --- | --- | --- |
| Go et al. (2011) | PWID (Male PWID aged 18-45), (299) | Community-based cross-sectional | Questionnaire survey;  Forward stepwise regression |
| Quan et al. (2009) | PWID (309) | (1) Cross-sectional  (2) Matched case-control study | Community-based survey;  Multivariate logistic regression models |
| Nguyen et al. (2007) | PWID (2023)  FSW (1391) | Cross-sectional | Survey;  Logistic regression models |
| Tran et al.(2006) | PWID (aged 14-29), (248) | Cross-sectional | Survey; Multivariate logistic regression |
| Nguyen et al. (2001a) | PWID (319) | Cross-sectional | Survey;  Stepwise logistic regression |
| Nguyen et al. (2001b) | PWID (518)  •On the street (218)  •In a rehab centre: (300) | Cross-sectional | Survey; Multivariate logistic regression |
| Nguyen et al. (2017) | FSW (aged18+) (420) | Community-based cross-sectional | Survey; Multivariate logistic regression |
| Le et al. (2015) | FSW (aged18+) (5298) | Cross-sectional | Survey; Multivariate logistic regression |
| Tran et al.(2014) | FSW (1996)  -Street-based (SSW): 339  -Establishment-based (ESW): 1657 | Cross-sectional | Survey; Multivariate logistic regression |
| Nguyen et al. (2009) | FSW (406)  *395 provided biological samples | Cross-sectional | Survey; Multivariate logistic regression |
| Thuong et al. (2005) | FSW (911) | Cross-sectional | Survey; Multivariate logistic regression |
| Tran et al.(2005) | FSW (400) | Cross-sectional | Survey; Multivariate logistic regression |
| Nguyen et al. (2004) | FSW (398) | Cross-sectional | Survey; Multivariate logistic regression |
| Nguyen et al. (2016) | MSM (2768) | Cross-sectional | Survey; Multivariate logistic regression (backward elimination method) |
| Le et al. (2016) | MSM (399) | Cross-sectional | National surveillance survey; multivariate logistic regression |
| Pham et al. (2012) | MSM (381):  PWID-MSM (63) and non-PWID-MSM (318) | Cross-sectional | Community based survey; multivariate Poisson regression (backward elimination) |
| Nguyen et al. (2008) | MSM (599) | Cross-sectional | Survey; multivariate logistic regression |

**Supplementary Table S4** Sociodemographic and sexual transmitted infections (STI), knowledge and perception associated with HIV infection in the included studies.

| **Authors/ year of publication** | **Sociodemographic risk factors** | **Associated STIs and knowledge/ perception of HIV** |
| --- | --- | --- |
| Go et al. (2011) | Age: (ref. 32+)  •23-26, OR: 4.66 (2.03, 10.72)  •27-31, OR: 3.64(1.62, 8.19) |  |
| Quan et al. (2009) | No significant variable found. |  |
| Nguyen et al. (2007) | PWID sample  •Unmarried: OR: 5.0, 95%CI not available  •Age: Young (15-24) (data not available)  •Higher education attainment  OR: data not shown  FSW sample  •Age: Young (15-24) (data not available) | PWID sample  •Knowledge of own HIV status  OR: 3.0, 95%CI not available  FSW sample  •Knowledge of own HIV status  OR<1, 95%CI not available |
| Tran et al.(2006) | •Age: Old (18-20), OR: 3.30 (1.09, 9.94)  •Education: Illiteracy/primary (compared with 10-12 years), OR: 2.37(1.02, 5.51); Secondary (6-9 years), OR: 2.07(1.02, 4.21) |  |
| Nguyen et al. (2001a) | No significant variable found. |  |
| Nguyen et al. (2001b) | •Marital status  Being divorced/separated (compared with single/married)  Street: OR 1.92 (1.04, 3.54)  Rehab centre: OR 2.05 (1.40, 4.06) |  |
| Nguyen et al. (2017) | •Income:  Low (≤ $200AUD), OR: 6.33 (1.66, 24.09) | •HIV Knowledge: (ref. High)  Low-medium 7.79 (7.07-56.59) |
| Le et al. (2015) | High HIV provinces  •Age: (ref. 18-24)  25-29, OR: 2.75 (1.72, 4.40)  30+, OR: 1.95 (1.20, 3.14)  • Marital status (ref. never married)  Widowed, OR: 1.75 (1.02, 3.00)  Low HIV provinces  •Marital status (ref. Never married):  Married, OR: 2.83 (1.48, 5.41)  Separated/divorced OR: 2.28 (1.30, 3.99)  Widowed, OR: 8.94 (4.54, 17.61) | Low HIV provinces  HIV knowledge:  • Correctly identified risks, OR: 1.51 (1.02, 2.25) |
| Tran et al. (2014) | •Number of clients: (ref. <16)  >16, OR: 2.65 (1.26-5.59) |  |
| Nguyen et al. (2009) | Age (a continuous variable):  •Older, OR: 1.1 (1.01, 1.26)  Age of first sex: (ref. >15)  •≤ 15, OR: 6.8 (1.07, 43.6) | Presence of STIs  •Trichomoniasis, OR: 11.7 (1.43-95.3)  •Candidiasis, OR: 15.4 (2.55-26.75) |
| Thuong et al. (2005) | •Income: (ref. High)  Low (<$33USD/month), OR: 2.36 (1.03, 5.37)  •Age of first sex:  ≤ 15, OR: 5.48 (1.67, 18.0) |  |
| Tran et al.(2005) | •Age: (ref. Old)  Young, OR: 1.2 (1.1, 1.4)  •Education: borderline significance  Low (≤ 7 years), OR: 3.4 (1.0, 11)  Income (self-reported, ref. Low):  • High, OR: 278 (9, 8465)  •Medium, OR: 10 (1.2, 82)  Length of residency in Hanoi  •Longer, OR: 1.3 (1.1, 1.6) | HIV risk perception (ref. No risk)  •Low, OR: 9.7 (2, 47)  HIV knowledge level (ref. High, score 9-10/10)  •Low (score ≤5 /10), OR: 4.3 (1, 19) |
| Nguyen et al. (2004) | •Age:  Young (<25), OR: 5.22 (2.10, 12.97) |  |
| Nguyen et al. (2016) | •Age: (continuous variable)  Risk increasing with age, OR: 1.13, (1.08, 1.18)  •Marital status: (ref. never)  Ever married, OR: 0.10 (0.03, 0.29)  •Having a religion:  Yes, OR: 3.56 (2.21, 5.73) | •HIV risk self-assessment (likelihood of infection)  -Likely, OR: 2.48 (1.00, 6.18)  -Very likely, OR: 3.76 (1.20, 11.9)  •Syphilis (+)  OR: 8.12 (2.59, 25.53) |
| Le et al. (2016) | •Age: (ref. <=25)  Older (than 25), OR: 7.82 (3.37, 18.16)  •Education: (ref. High)  Low (< Year 5), OR: 2.74 (1.10, 6.83) | •HIV risk self-assessment (ref. No):  Yes (at-risk), OR: 2.42 (1.24, 4.73) |
| Pham et al. (2012) | •Gender identity/sexual orientation (ref. homosexual)  Transgender, OR: 4.27 (1.17, 15.57) |  |
| Nguyen et al. (2008) | •19<Age<41 (ref. older), OR: 4.43 (1.45-13.59)  •Low education (< 6 years), OR: 2.60 (1.32-5.13) |  |

**Note:** Ref.: Assigned reference group in the identified variable (in logistic/ multivariate regression result)

**Results presented in Supplemental Table S4**

Four PWID and FSW studies (8, 21, 22, 26) revealed that low levels of HIV knowledge increased the risk of HIV infection. On the other hand, self-perceived risk of HIV was found to be a positive predictor of HIV prevalence in one FSW publication (26) and two MSM studies (7, 28). In addition, sociodemographic characteristics such as age, marital status, education and income were commonly reported by many studies. The results of systematic review of these variables are displayed in Supplementary Table S4. Due to varying definitions and measurements of these variables across the selected studies, meta-analyses on these factors could not be performed to estimate the prevalence of these factors.

**Sociodemographic risk factors**

The factor with the greatest number of significant reported associations in all the categories

was the demographic factor of age. Ten of the seventeen (58.82%) studies identified age as a significant risk factor. Of the ten, five studies associated being older with HIV and the other half suggested younger age as a risk factor. For example, two MSM studies (7, 28) showed that older age was associated with HIV infection. However, the other MSM study, Nguyen et al. (29) found that being younger the age of 41 had a strong association (OR: 4.43) with HIV infection. Similarly, two PWID studies (8, 16) identified younger age a risk factor, while one PWID study (18) found that older age (> age 17) increased the risk of HIV. Inconsistent findings regarding the association between age and HIV were also demonstrated in FSW studies. As the age cut-offs used to determine young or old age groups (some defined age as a continuous variable) differed greatly among the included studies, meta-analysis could not be performed to estimate the pooled effect.

Four studies (7, 17, 18, 26) representing three at-risk groups found that lower levels of education was significantly associated with HIV infection (OR value ranging from 2.6 to 3.4). However, Nguyen et al.(8) showed that higher education attainment increased the odds of HIV infection. These studies defined ‘low education’ differently, using a cut-off point at Year 5, 6, 7 or high school level (secondary school). Similarly, inconsistent definition of income level was also observed in our systematic review. Nguyen et al. (21) used less than $200AUD per month and Thuong et al. (25) used less than $33USD per month as the ‘low income’ cut-offs. Both studies showed positive associations between low income and HIV prevalence. In contrast, Tran et al. (26) applying self-reported income level in comparison with peers, found that higher income appeared to increase the likelihood of infection. Due to the varying definitions of income levels used in these studies, it is difficult to draw any conclusion on the effect of income on HIV among FSW.

The impact of marital status was identified by four studies. Nguyen et al. (8) with PWID and Nguyen et al. (28) with MSM suggested never married as a risk factor of HIV, whereas Le et al. (22) with FSW showed that ever being married (including being married, separated or widowed) increased the odds of HIV infection compared to those never married. On the other hand, Nguyen and colleagues (20) reported that being divorced or separated was more likely to be HIV positive than single and married PWID participants. In addition, two FSW studies (24, 25) reported a strong association between the age of first sex being equal to or below 15 and higher HIV prevalence. Both studies used identical definitions of the risk factors reported ORs 6.8 and 5.48 (95%CI: 1.07-43.6 and 1.67-18.0) respectively. Based on the results of these two studies, the age of first sex being below 15 appeared to be associated with HIV. Meta-analysis could not be performed as these factors were defined differently (using varying cut-off points to determine high or low levels) in the included studies or there was an insufficient number of studies for the analysis of each demographic variable.

**Supplementary Figure S1 Estimated prevalence of injecting drug use**

**A. Injecting drug use among FSW**


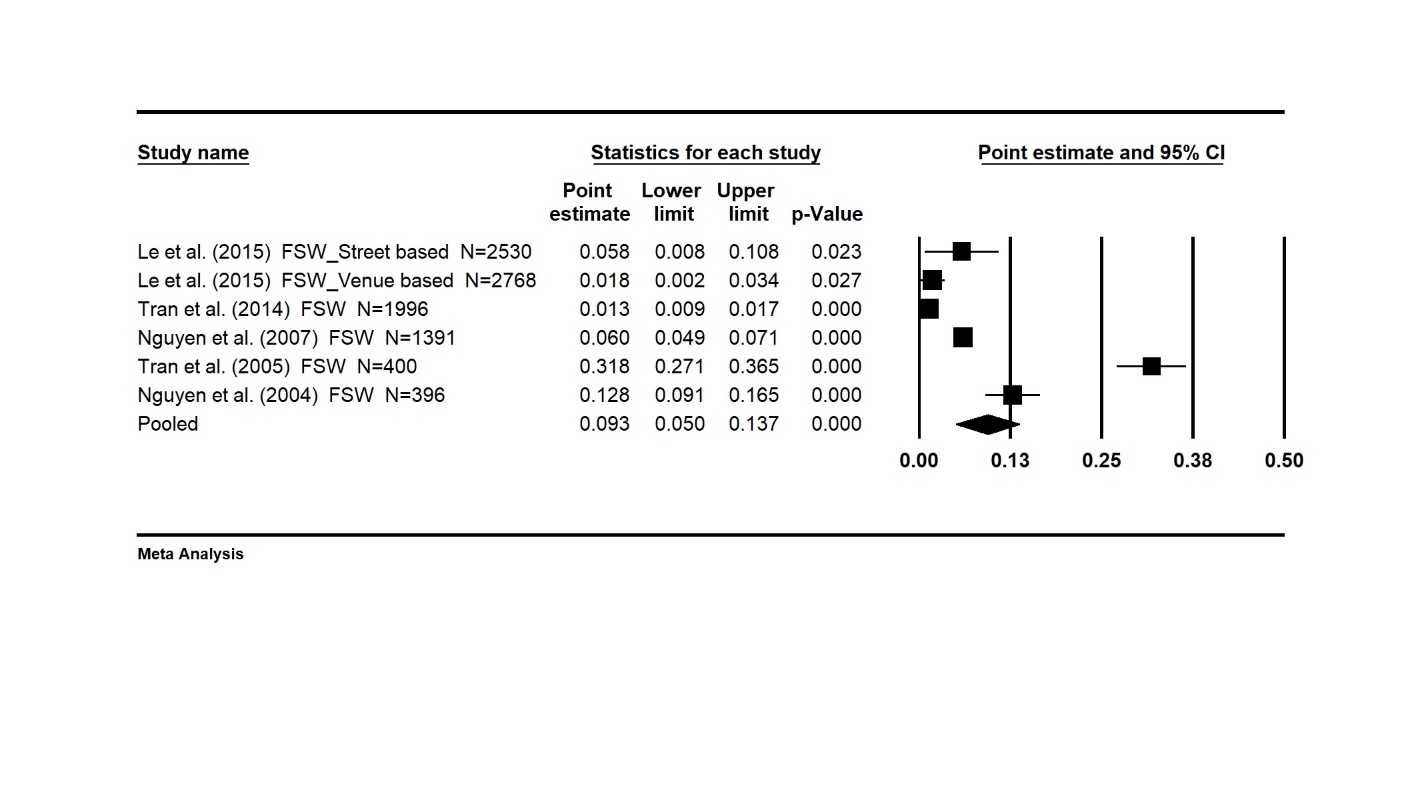


**B. Injecting drug use among MSM**


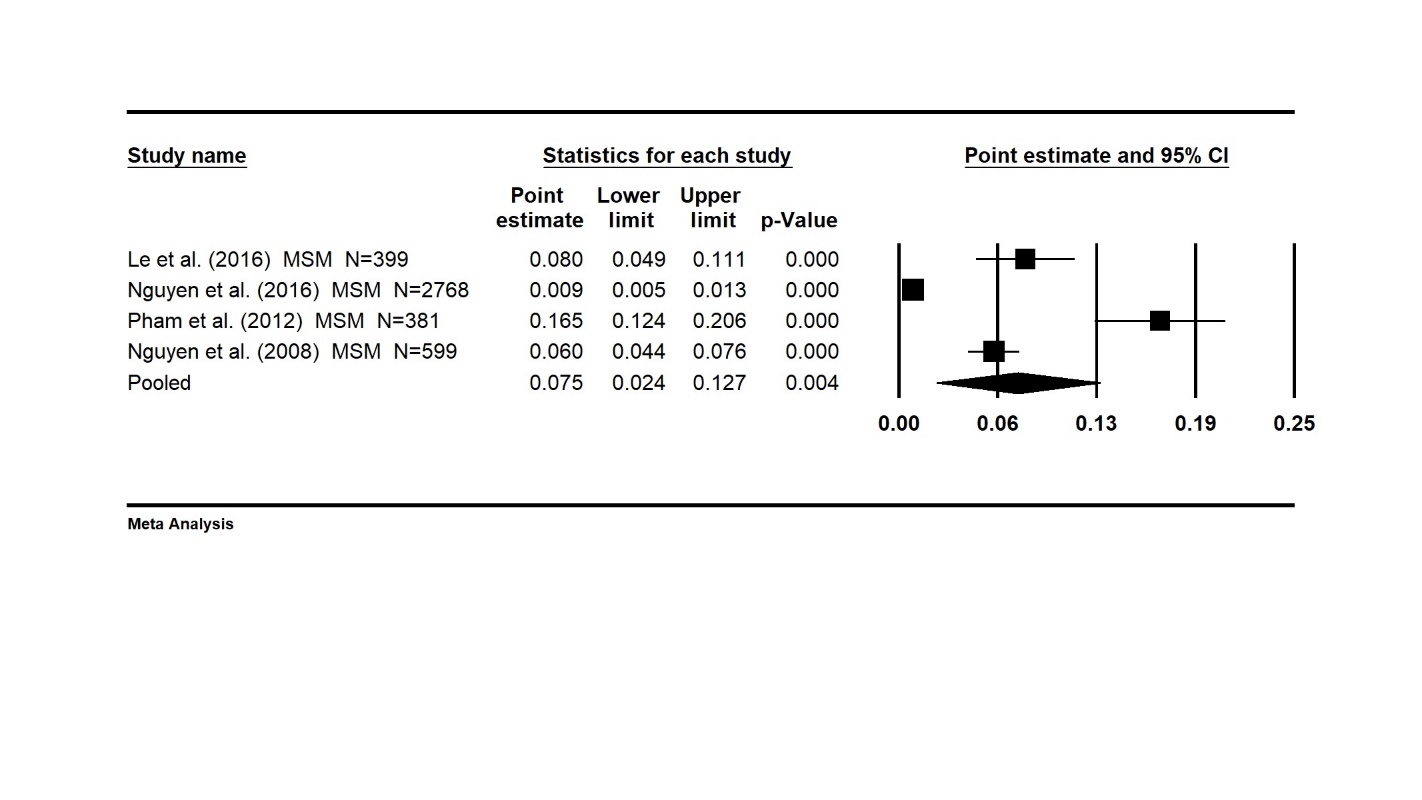


**Note:** 1. The size of each box is proportional to the weight (sample size) of the study and the horizontal line through each box represents the 95% confidence intervals (95%CI) for the study measure; 2. The diamond shape represents the pooled effect size (odds ratio) of the meta-analysis. The centre of the diamond is the point estimate, and the line ends reflect the 95%CI; 3. FSW_Street-based and FSW_Venue-based are street-based and venue-based FSW subsamples.

**Supplementary Figure S2 Estimated prevalence of multiperson use of injecting equipment**

1. **Multiperson use of injecting equipment among PWID**


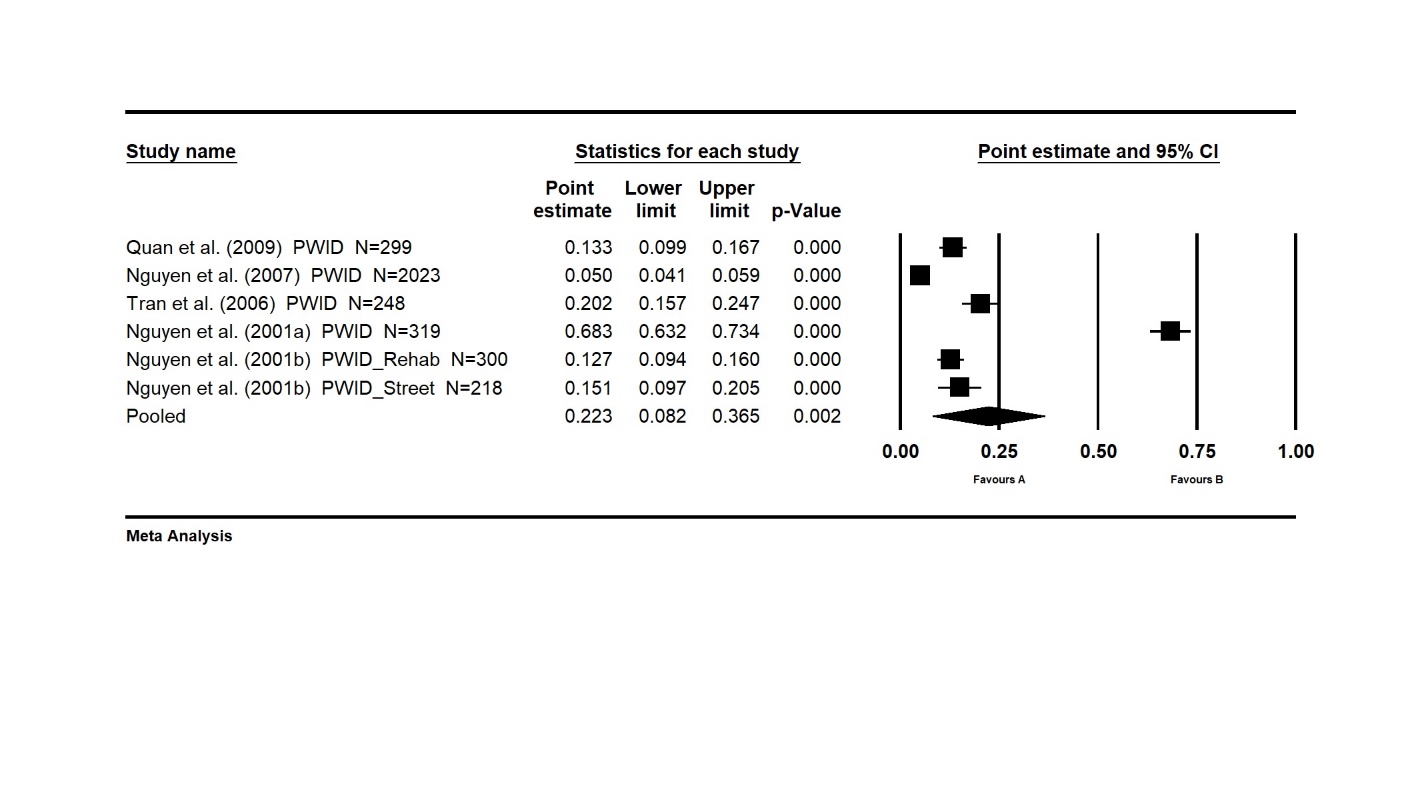


1. **Multiperson use of injecting equipment among PWID and FSWs**


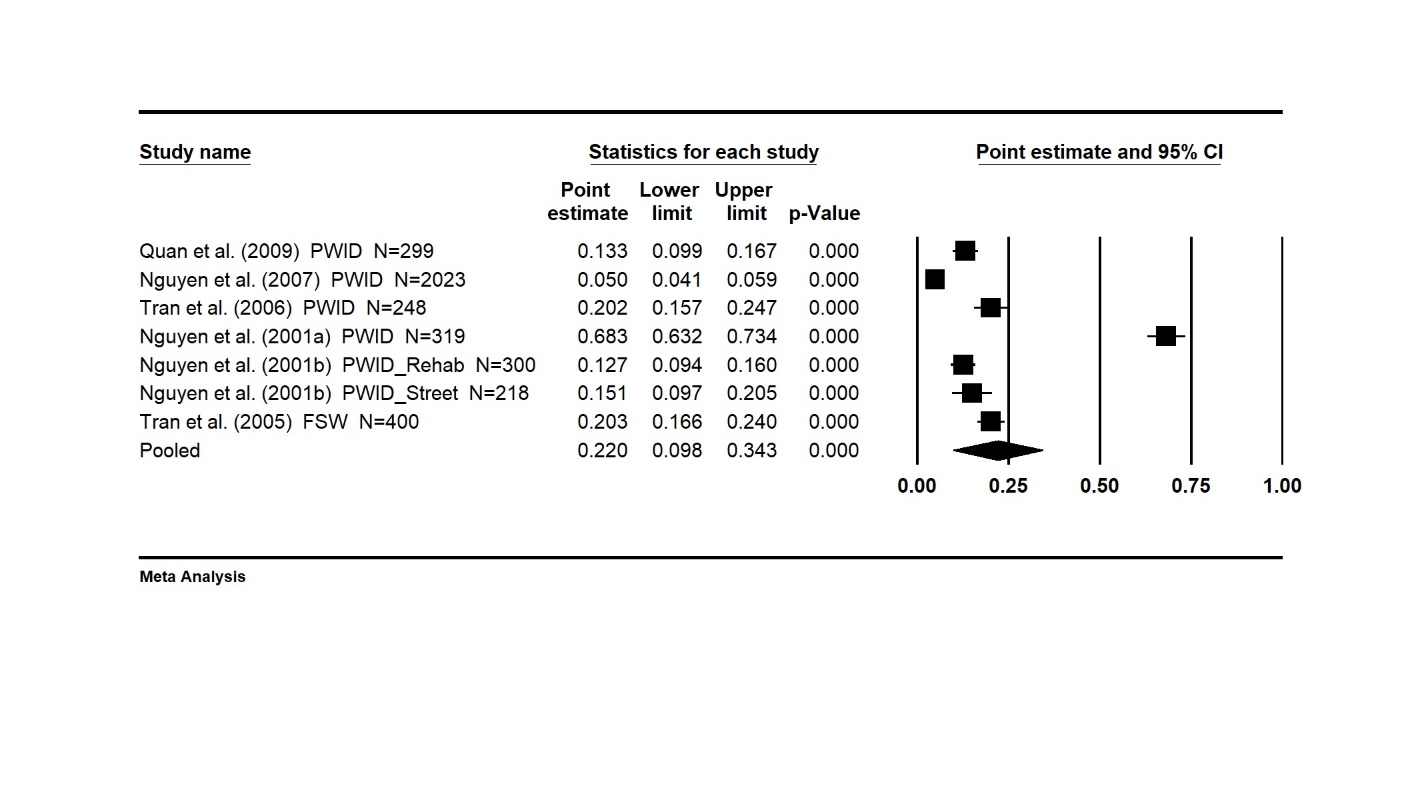


**Note:** 1. The size of each box is proportional to the weight (sample size) of the study and the horizontal line through each box represents the 95% confidence intervals (95%CI) for the study measure; 2. The diamond shape represents the pooled effect size (odds ratio) of the meta-analysis. The centre of the diamond is the point estimate, and the line ends reflect the 95%CI; 3. PWID_Rehab and PWID_Street are subsamples recruited in a large rehabilitation centre in HCMC and in the street.

**Supplementary Figure S3 Estimated prevalence of inconsistent condom use**

**A. Inconsistent condom use among PWID**


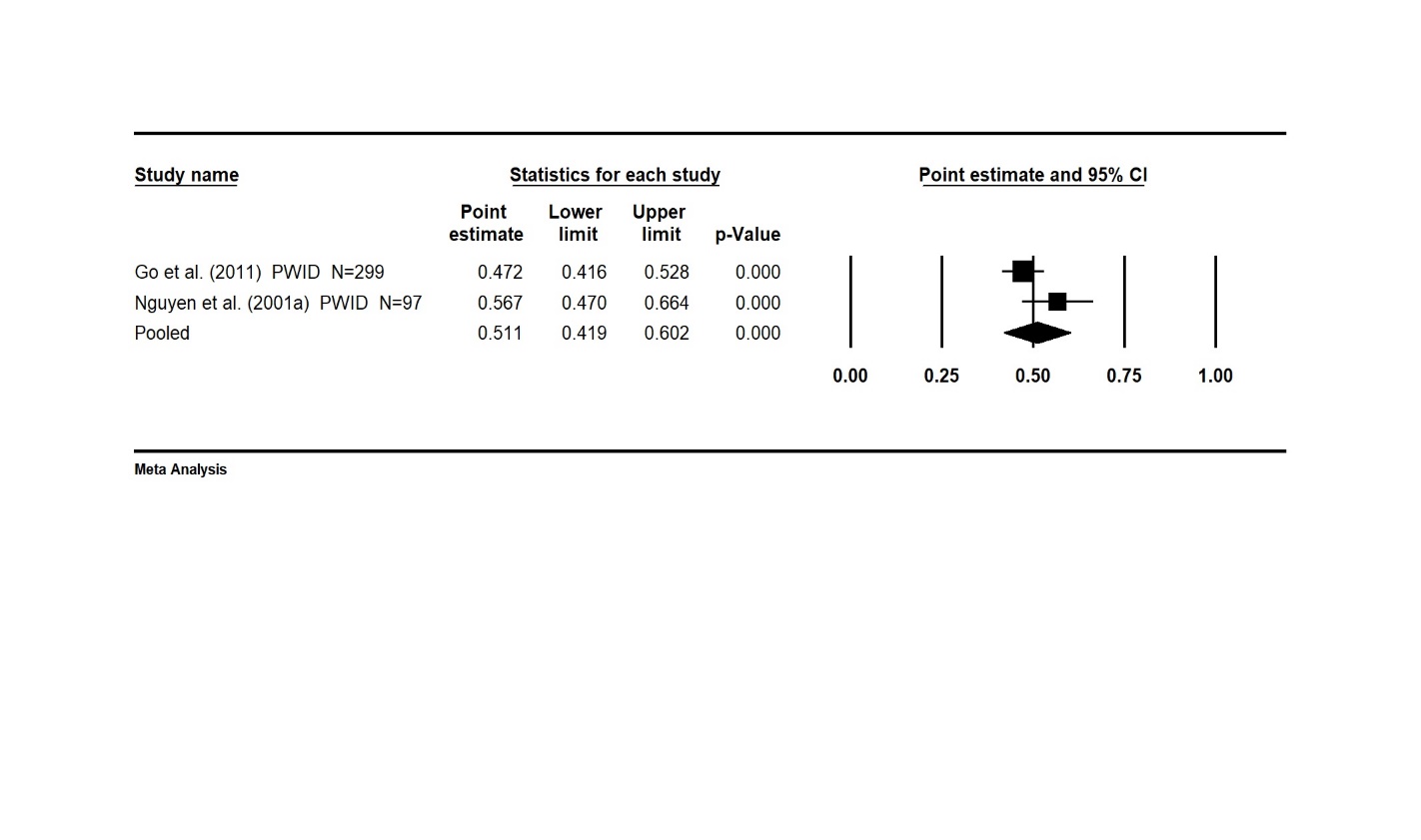


**B. Inconsistent condom use among FSW**


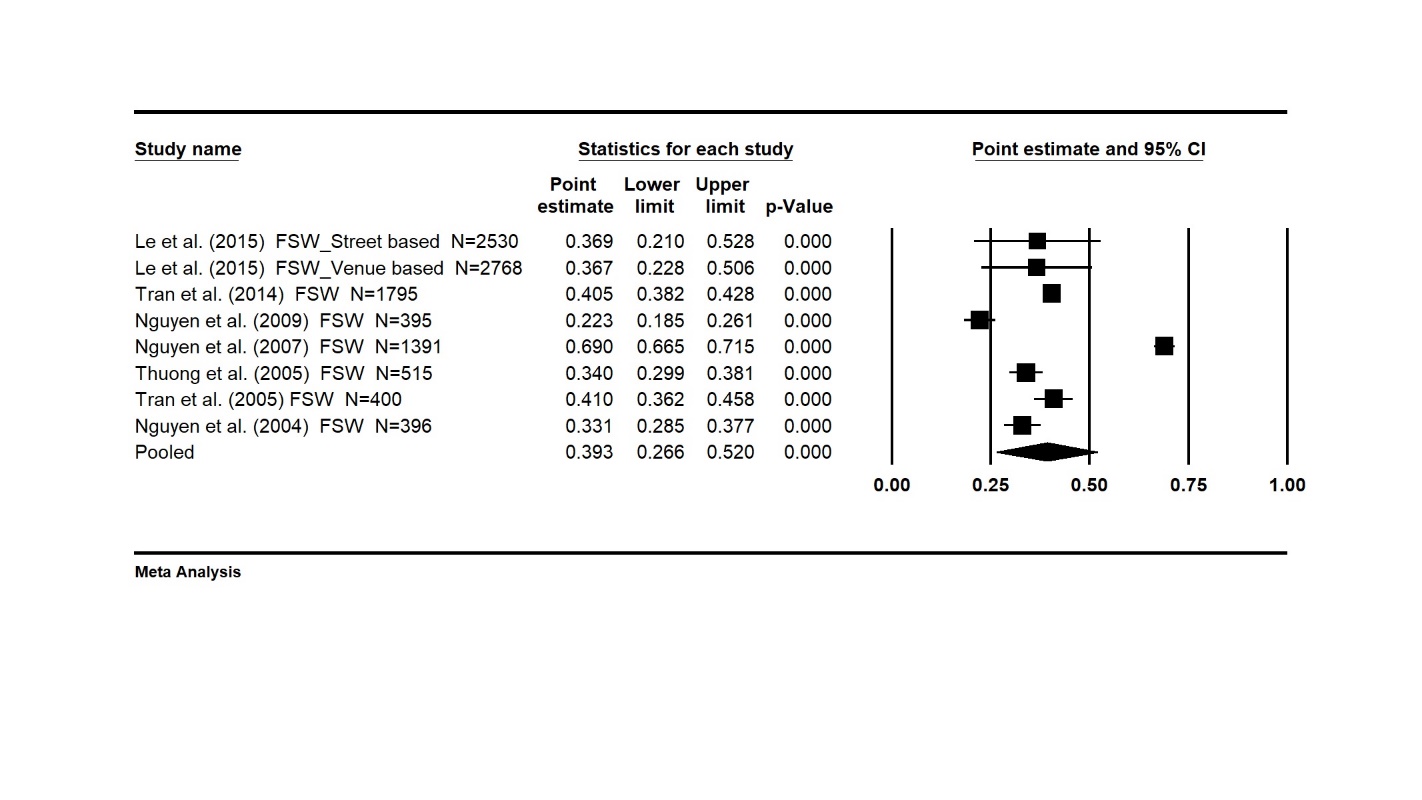


**C. Inconsistent condom use among MSM**


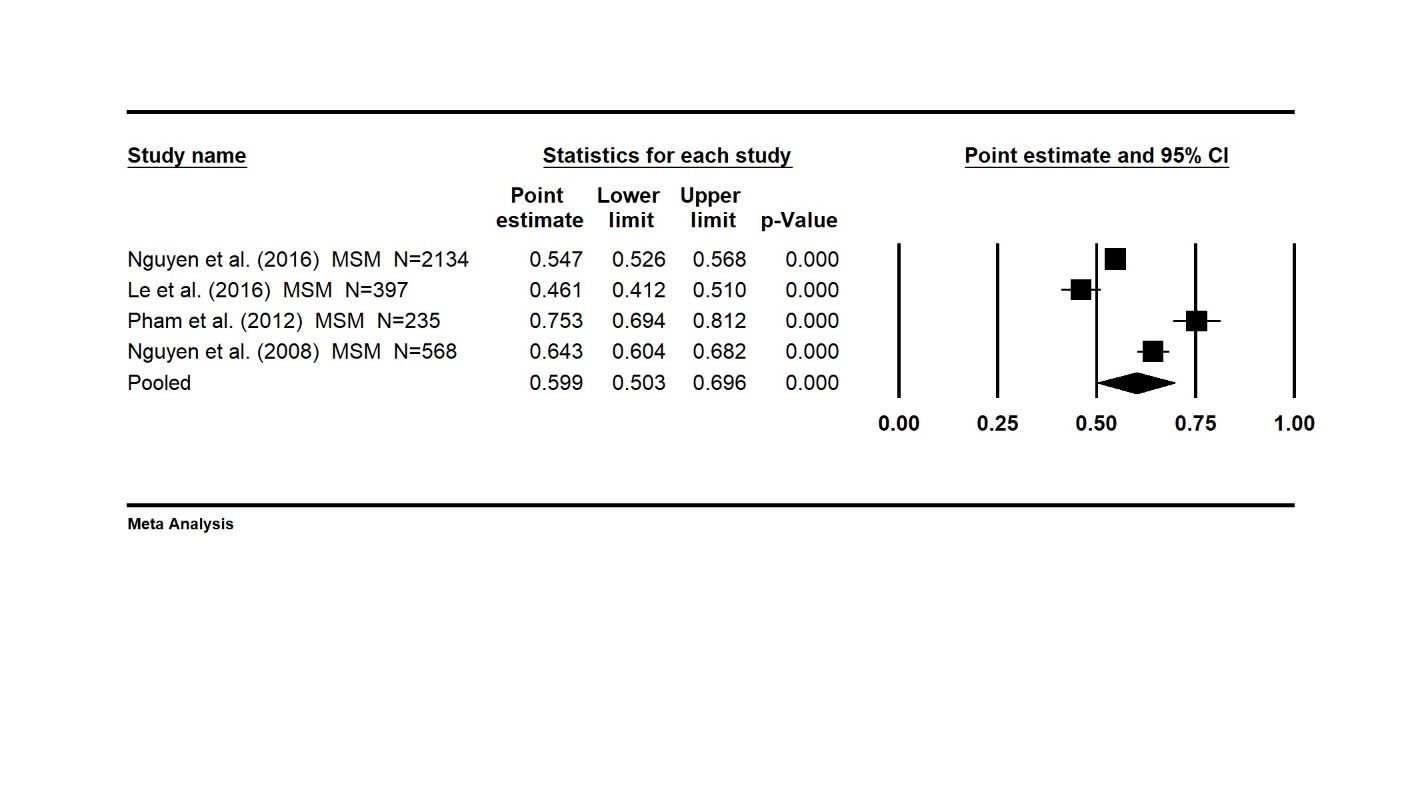


**Note:** 1. The size of each box is proportional to the weight (sample size) of the study and the horizontal line through each box represents the 95% confidence intervals (95%CI) for the study measure; 2. The diamond shape represents the pooled effect size (odds ratio) of the meta-analysis. The centre of the diamond is the point estimate, and the line ends reflect the 95%CI; 3. FSW_Street-based and FSW_Venue-based are street-based and venue-based FSW subsamples.
